# Towards Using the Instrumented Timed Up-and-Go Test for Screening of Sensory System Performance for Balance Control in Older Adults

**DOI:** 10.3390/s19030622

**Published:** 2019-02-01

**Authors:** Thomas Gerhardy, Katharina Gordt, Carl-Philipp Jansen, Michael Schwenk

**Affiliations:** 1 Network Aging Research, Heidelberg University, Bergheimer Strasse 20, 69115 Heidelberg, Germany; gerhardy@nar.uni-heidelberg.de (T.G.); gordt@nar.uni-heidelberg.de (K.G.); jansen@nar.uni-heidelberg.de (C.-P.J.); 2 Institute of Sports and Sports Sciences, Heidelberg University, 69120 Heidelberg, Germany

**Keywords:** Timed Up-and-Go, mobile sensing, screening, mobility

## Abstract

*Background*: Decreasing performance of the sensory systems’ for balance control, including the visual, somatosensory and vestibular system, is associated with increased fall risk in older adults. A smartphone-based version of the Timed Up-and-Go (mTUG) may allow screening sensory balance impairments through mTUG subphases. The association between mTUG subphases and sensory system performance is examined. *Methods*: Functional mobility of forty-one community-dwelling older adults (>55 years) was measured using a validated mTUG. Duration of mTUG and its subphases ‘sit-to-walk’, ‘walking’, ‘turning’, ‘turn-to-sit’ and ‘sit-down’ were extracted. Sensory systems’ performance was quantified by validated posturography during standing (30 s) under different conditions. Visual, somatosensory and vestibular control ratios (CR) were calculated from posturography and correlated with mTUG subphases. *Results*: Vestibular CR correlated with mTUG total time (r = 0.54; p < 0.01), subphases ‘walking’ (r = 0.56; p < 0.01), and ‘turning’ (r = 0.43; p = 0.01). Somatosensory CR correlated with mTUG total time (r = 0.52; p = 0.01), subphases ‘walking’ (r = 0.52; p < 0.01) and ‘turning’ (r = 0.44; p < 0.01). *Conclusions*: Supporting the proposed approach, results indicate an association between specific mTUG subphases and sensory system performance. mTUG subphases ‘walking’ and ‘turning’ may allow screening for sensory system deterioration. This is a first step towards an objective, detailed and expeditious balance control assessment, however needing validation in a larger study.

## 1. Introduction

Age-associated deterioration in balance abilities often results in falls which have a strong impact on older people’s quality of life [1]. For an efficient performance in day-to-day motor tasks, control of postural stability is crucial. A well-functioning sensory system is required to compensate for permanent destabilization during human movement. Natural aging processes and age-related diseases can affect different sensory systems relevant for balance control including the visual, vestibular and somatosensory system [2,3]. Visual perception usually starts to decline at the age of fifty in terms of impaired depth perception and reduced contrast sensitivity [4]. Aging also affects the vestibular system and reduces the number of receptors in the vestibular organ [5]. Golder et al. [6] showed that older subjects with impaired vestibular function had significantly poorer functional mobility than non-impaired peers. In old age, common chronic illnesses often lead to a degenerated nervous system [7] which may affect the somatosensory system as well as the proprioception [8,9]. 

So far, accurate and objective assessment of sensory systems relevant for balance control is complex, time consuming and requires specific equipment [10]. Subjective assessments such as the Clinical Test for Sensory Interaction on Balance (CTSIB) require trained investigators and are not part of the standard mobility assessment in geriatric patients [11]. The CTSIB is recommended as a screening test for sensory system performance of neurological patients [12], but the scaling has limitations regarding discrimination between different patient groups. Posturography for example has to be performed by medical professionals and is not applicable in geriatric screenings as well as examinations of visual impairment. Additionally, clinical balance assessments are prone to ceiling effects [13]. Despite the importance of sensory system function for balance control, a quick objective screening tool for testing sensory systems’ performance during functional mobility tasks does not exist to the best of our knowledge.

One of the most commonly used tools for measuring functional mobility is the Timed Up-and-Go (TUG). The conventional TUG provides a general estimation of functional mobility based on the duration to perform the test, usually measured by using a stopwatch [14]. While the TUG estimates global functional mobility, it does not provide information on underlying sensory deficits, which have an influence on functional performance. Moreover, TUG results do not allow differentiation between subjects with lower performance in turning, walking or other subphases within the TUG. In recent years advances in wearable sensor technology have provided a new chance for measuring several subphases of the instrumented TUG including rising from a chair, walking, turning and sitting down [15]. Using sensor systems while screening balance ability could help to make more precise predictions about the functioning of the postural system [15] and the quality of the movement execution [16]. The technical progress using wearable sensors—so called inertial measurement units (IMU)—has the potential to improve the quality of spatial and temporal movement tests [17]. Recent advances in these clinically approved tests can provide useful additional information during usual assessment without previous technical support. By now, the complexity of the TUG with its different subphases walking, turning, rising from a chair and sitting down has not been used for deeper examination of functional performance. With this, deficits can be refined to individual subphases of the TUG and a more exact statement about the functional mobility becomes possible [15]. A previous study showed that turning might be the most challenging movement task within the TUG, and for Parkinson’s disease patients turning seems to be the most sensitive deficit to detect impairments [18]. IMUs and smartphones seem to be a reasonable choice for measuring functional mobility, considering their wide distribution and easy handling for untrained persons. In this context, performance in each of the TUG subphases may also provide meaningful information about the performance of the sensory system related to balance control. It gives the opportunity to quantify balance deficiencies, which would remain undetected using total duration as derived from the traditional TUG version. However, to the best of our knowledge, no study has explored this potential association. Consequently, the aim of this study was to evaluate the association between TUG subphases and performance of the sensory system in an explorative sample. 

## 2. Materials and Methods

### 2.1. Study design and participants

In this cross-sectional study, subjects were recruited through direct contact at university events (open day and seminars at the Network Aging Research, Heidelberg University, Heidelberg, Germany), via the outpatient geriatric rehabilitation unit at AGAPLESION Bethanien Hospital, Heidelberg, and brochures displayed at both institutes. Subjects were included if they were aged 55 years or older, able to walk independently for ten meters, and willing to sign a written informed consent. An age of 55 years or older was chosen as inclusion criterion based on literature showing that accelerated sensory performance decline starts to occur above this age [19,20]. Subjects were excluded if they had serious neurological, cardiovascular or cardiopulmonary diseases, visual impairment which could not be compensated with glasses, and if they showed signs of cognitive impairment (Mini Mental State Examination score, MMSE <26) [21]. Ethical approval was obtained from the local institutional review board at Heidelberg University and is in agreement with the Declaration of Helsinki. All participants provided written informed consent prior to participation.

### 2.2. Measures

Participant characteristics including age, gender, comorbidities, living situation and falls in the past year were collected by questionnaire administered by an assessor. Cognitive status was assessed using the MMSE [22]; then, fear of falling was assessed using the Short Falls Efficacy Scale-International (Short FES-I) [23], with a score >10 indicating a high fear of falling. Overall balance performance was assessed using the Berg Balance Scale (BBS) [24] with a score <45 indicating an increased fall risk.

### 2.3. mTUG 

In this study, an instrumented TUG (mTUG, mHealth Technologies, Bologna, Italy) was performed with a smartphone (Samsung Galaxy S4, Samsung Group Seoul, Japan, Android 5.0.1) fixed on the lower back using an elastic belt. In a previous study, algorithms of the system were validated with a McRoberts Dynaport Hybrid, which showed that the mTUG system is capable of measuring movement tasks [25]. Instrumented TUG-testing allows discrimination of subphases based on smartphone-integrated sensor data, like accelerometer, gyroscope and magnetometer.

The mTUG procedure was identical to the usual TUG, that is, stand up from a chair, walk three meters in comfortable pace, turn around, walk back and sit down. mTUG trials were analyzed using a validated smartphone application described elsewhere [25]. In brief, acceleration and gyroscope signals were analyzed by validated algorithms which automatically detect mTUG subphases ’sit-to-walk’, ‘walking’, ‘turning’, ‘turn-to-sit’ and ‘sit-down’ [25]. The mTUG total time and split-time of each subphase were used for analysis. The mTUG was performed three times. The first trial was a test trial to ensure accordance with the testing procedure. Mean values of the second and third trial were used for analyses.

### 2.4. Sensory Analysis

Sensory Analysis (SA), a modification of the CTSIB, included a series of four static standing tasks with modified vision (eyes open – eyes closed) and somatosensory inputs (floor – foam) [26]. Trial duration for each test was 30 s, with subjects’ feet placed shoulder-width apart and arms loose on their side. During standing tasks, subjects wore an IMU (Valedo, Hocoma AG, Volketswil, Switzerland) at the lumbar spine, which recorded three dimensional sway. The angular velocity range (v) in anterior-posterior direction was used for analyses, as it is most affected by a balance deficit during stance [26]. A formula was used to calculate the Control Ratios (CR) which describe the sensory input of each sensory system (vi = visual system; s = sensory system; ve = vestibular system) to balance control [26]. More specifically, the increased anterior-posterior velocity range during, e.g. eyes closed compared to eyes open tasks, was set in proportion to the anterior-posterior angular velocity range during all tasks. Higher scores indicate a higher proportion of sensory input of the respective sensory system. The CR was calculated as follows:

$${\mathrm{CR}}_{\mathrm{vi}}=\left(\frac{\left(v_{ecf}-v_{eof}\right)+\left(v_{ec}-v_{eo}\right)}{\left(v_{\mathrm{eo}}+v_{\mathrm{ec}}+v_{\mathrm{eof}}+v_{\mathrm{ecf}}\right)}\right)\;\times \;100\;{\mathrm{CR}}_{s}=\left(\frac{\left(v_{ecf}-v_{\mathrm{ec}}\right)+\left(v_{\mathrm{eof}}-v_{\mathrm{eo}}\right)}{\left(v_{\mathrm{eo}}+v_{\mathrm{ec}}+v_{\mathrm{eof}}+v_{\mathrm{ecf}}\right)}\right)\times 100\;{\mathrm{CR}}_{\mathrm{ve}}=100-{\mathrm{CR}}_{\mathrm{vi}}-{\mathrm{CR}}_{s}$$

where CR_vi_: visual control ratio; CR_s_: somatosensory control ratio; CR_ve_: vestibular control ratio; ec: eyes closed; eo: eyes open; f: foam

### 2.5. Statistical Analysis

Data were summarized as mean and standard deviation (SD) for continuous measures and number and percentage for dichotomous measures. Pearson’s correlation coefficients were calculated between mTUG total time and each CR, as well as between each mTUG subphase and each CR. Pearson’s correlation coefficients of 0.10–0.30 were classified as small, 0.30–0.50 as medium, and ≥0.50 as large [27]. Statistical analysis was carried out using SPSS 24 (IBM, Armonk, NY, USA).

## 3. Results

Nine male and 32 female subjects between 58 and 89 years of age (mean: 72.6 ± 7.5 years) took part in the study. All participants were living independently at home, either alone or with their spouse. Participant characteristics are shown in Table 1. Ten participants (24.4%) reported one or more falls within the last year and 23 subjects (56.1%) reported at least one comorbidity. Fear of falling (Short FES-I) was high with a mean score of 10.2 ± 2.9 [28]. BBS score was high with 50.8 ± 8.1 points, which is above the cut-off value for risk of falling (<45 [24]). 

### 3.1. mTUG

mTUG results including subphase durations are illustrated in Table 2. The mean ‘total time’ for the mTUG was 12.97 ± 6.01 s. The longest duration was required for the subphase ‘walking’ with 6.71 ± 3.97 s; the shortest duration for ‘sit down’ 1.57 ± 0.68 s. SD of subphases ranged between 0.60 s for ‘sit-to-walk’ and 3.97 s for ‘walking’.

### 3.2. Sensory Analysis 

Table 3 shows the anterior-posterior sway (m/s^2^) during the single stance tasks of the SA. Descriptive results showed that tasks with eyes closed resulted in increased sway compared to the same tasks with eyes open. Likewise, sway was more affected by closed eyes (5.05 ± 3.29 m/s²) than by standing on foam with eyes open (4.84 ± 1.99 m/s²). The combination of closed eyes and standing on foam resulted in the largest anterior-posterior sway (7.87 ± 7.84 m/s²).

### 3.3. Control Ratio

CR were calculated for anterior-posterior sway (Table 4), using the formula by Hegemann et al. [26]. Vestibular CR showed the highest score (78.01 ± 28.20%), visual CR had the lowest score with 9.23 ± 14.61% and somatosensory had a slightly higher contribution with 12.62 ± 20.46%.

### 3.4. Correlation of CR with mTUG

Correlation of CR with mTUG total time and subphases are shown in Table 5. Medium to large correlations are shown with all mTUG subphases and vestibular CR (r = −0.31–0.56; p = <0.01–0.05). Somatosensory CR had significant correlations with all mTUG subphases (r = 0.41–0.52; p = 0.01), except ‘sit-to-walk’ (r = 0.19; p = 0.23). Visual CR had medium correlations with mTUG ‘total time’ (r = 0.32; p = 0.04), ‘sit-to-walk’ (r = 0.36; p = 0.04) and ‘walking’ (r = 0.34; p = 0.03).

## 4. Discussion

This study aimed to evaluate the association between mTUG subphases and performance of sensory systems which are relevant for balance control. Results indicate that specific sensory system performance was linked to specific functional mobility components quantified by mTUG subphases. mTUG total time was most strongly associated with vestibular and somatosensory system performance. This is in line with a previous study, which showed that these systems are mainly responsible for balance control and have a strong influence on functional mobility [6,29]. Golder et al. [6] could show that vestibular sensory system performance measured by the CTSIB explained nearly 50% of the variance in total TUG time performance. However, Golder et al. used an observer-based assessment (CTSIB) for sensory system performance, which is prone to ceiling effects and observer bias. Apart from demonstrating the general relationship between sensory system performance and stopwatch-recorded total TUG time, Golder et al. did not provide insights about the relationship between specific TUG subphases and sensory system performance. Previous studies have shown that the specific subphase ‘turning’ is superior to total TUG time with respect to certain outcomes. For example, turning had the highest accuracy for discriminating healthy from Parkinson’s disease patients [18]. Building up on the approach by Golder et al., the present approach is a step forward by applying an objective, instrumented assessment method allowing insight into the interplay between TUG subphases and sensory system performance. Our results highlight that a lower vestibular subsystem performance specifically impacts on the mTUG suphases of ‘turning’ and ‘walking’ whereas other subphases are less affected. Our findings are in line with previous studies demonstrating that vestibular function controls postural stability during walking and turning [30]. 

Our findings demonstrate the proof-of-concept to use the mTUG subphases as a screening tool for deficits in clinically relevant sensory system performance, impacting on everyday mobility performances which are required for safe and independent ambulation [3]. The results are also in line with a recent study of Hafström et al. [31], which demonstrated that an impaired somatosensory system impacts on postural stability and balance control. 

Lower correlations were found between sensory CRs and subphases ‘sit-to-walk’, ‘sit-down’ and ‘turn-to-sit’. One possible explanation is that for these subphases lower limb strength is more important than sensory system performance as shown in previous studies [32,33]. In addition, visual CR showed lower correlations with mTUG ‘total time’ and the different subphases than the vestibular and somatosensory CR. The highest correlation between visual CR and TUG subphases was found for the ‘sit-to-walk’ subphase. We speculate that visual system performance could be relevant to prepare for the upcoming walking and turning tasks as well as for analyzing the predefined track, surroundings and surface conditions. 

Sensory system performance can be measured with several methods [34,35]. However, the time required for these assessments as well as the specifically trained personnel and the equipment hamper their application as screening tests in geriatric routine care. In contrast, our proposed mTUG approach for screening sensory subsystem performance simply requires a smartphone and can be implemented in one of the most widely used assessment procedures in routine care (TUG) without any additional time required for the assessment. Using objective assessments for evaluation of the sensory subsystem and mTUG performance makes our results robust against observer bias. Hence, our approach may have additional value for a more precise routine care assessment of functional performance and underlying deficits. On the same note, it can be implemented in a busy clinical setting without requiring additional time or specific training for observers.

## 5. Limitations

We recruited a convenient sample of community-dwelling, relatively fit older adults. Study participants’ BBS score (50.8 points) was above the cut-off value for increased fall risk (<45 [24]). While the sample may not represent the typical population for fall risk screening, we believe it was sufficient to demonstrate the proof-of-concept of our approach. The age cut-off used as an inclusion criterion in this study (≥ 55 years) is below the established cut-off for defining older adults (≥ 65 years). Therefore, our results may not be generalizable to the specific population of older adults aged ≥ 65 years. Studies with larger samples are required to validate our proposed approach in more impaired subjects and specific patient populations and age groups.

## 6. Conclusions

Our proposed mTUG analysis may allow for an objective, quick screening for underlying sensory deficits related to clinically relevant impairments in functional performances, which is easy to administer in clinical practice. This provides an opportunity for large-scale implementation without requiring much additional resources. Further studies are needed to validate the concept and establish normative values for a quick and easy interpretation of mTUG results.

## Figures and Tables

**Table 1:** Participant characteristics.

	Participants (*n* = 41)
Age (years), mean ± SD	72.6 ± 7.5
Women, % (n)	78.1 (32)
Participant with ≥ 1 fall in the past year, % (n)	24.4 (10)
Comorbidity prevalence (number), mean ± SD	0.95 ± 1.24
History of a stroke > 6 months ago (number)	6
Polyneuropathy (number)	3
Diabetes (number)	6
Non-acute cancer (number)	9
Controlled coronary heart disease (number)	8
Controlled pulmonary disease (number)	7
Mini Mental State Examination Test, mean ± SD (0–30 points)	28.9 ± 1.4
Short Falls Efficacy Scale-International, mean ± SD (7–28 points)	10.2 ± 2.9
Berg Balance Scale, mean ± SD (0-56 points)	50.8 ± 8.1

**Table 2:** mTUG subphases in seconds.

	Mean	±SD	Minimum	Maximum
mTUG total	12.97	6.01	6.83	35.06
sit-to-walk	1.75	0.60	0.38	4.56
walking	6.71	3.97	2.84	20.28
turning	1.61	0.71	0.82	4.34
turn-to-sit	2.85	1.40	1.65	8.41
sit down	1.57	0.68	0.87	3.93

**Table 3:** Sensory Analysis (SA); anterior-posterior sway during the SA during single stance tests in m/s².

		Mean	±SD	Minimum	Maximum
Firm surface	Eyes open	4.50	2.12	2.05	9.80
	Eyes closed	5.05	3.29	1.95	17.80
Foam surface	Eyes open	4.84	1.99	2.35	10.15
	Eyes closed	7.87	7.84	2.40	42.30

**Table 4:** Control Ratio (CR) of visual, somatosensory, vestibular contribution given as percentage; negative ratios are due to calculation by the formula by Hegemann et al. [26]; negative or ratios greater than 100% are due to higher values with eyes open vs eyes closed and, respectively floor vs foam surface, respectively.

	Mean	±SD	Minimum	Maximum
CR_visual_	9.23	14.61	−17.33	58.58
CR_somatosensory_	12.62	20.46	−22.67	79.80
CR_vestibular_	78.01	28.20	8.16	115.19

**Table 5:** Correlation of mTUG total time and mTUG subphases with CR, r = pearson correlation; CI: confidence interval.

	CR_Visual_			CR_Somatosensory_			CR_Vestibular_		
mTUG	r	95% CI	p-value	r	95% CI	p-value	r	95% CI	p-value
Total time	0.32	0.01–0.57	0.04	0.52	0.25–0.71	0.01	0.54	0.28–0.73	<0.01
Sit-to-walk	0.36	0.06–0.60	0.04	0.19	−0.13–0.47	0.23	−0.31	0.01–0.56	0.05
Walking	0.34	0.04–0.59	0.03	0.52	0.25–0.71	0.01	0.56	0.31–0.74	<0.01
Turning	0.21	−0.10–0.49	0.17	0.44	0.15–0.66	0.01	0.43	0.14–0.65	0.01
Turn-to-sit	0.14	−0.18–0.43	0.38	0.41	0.12–0.64	0.01	0.37	0.07–0.61	0.02
Sit-down	0.17	−0.15–0.45	0.30	0.42	0.13–0.64	0.01	0.39	0.09–0.62	0.01
